# Grain Growth upon Annealing and Its Influence on Biodegradation Rate for Pure Iron

**DOI:** 10.3390/ma15228030

**Published:** 2022-11-14

**Authors:** Yu Zhang, Ke Zhang, Weidong Liu, Zhongren Zheng, Mingchun Zhao

**Affiliations:** 1 Xiangya Hospital, Central South University, Changsha 410008, China; 2 National Clinical Research Center for Geriatric Disorders, Xiangya Hospital, Changsha 410008, China; 3 International Joint Research Center of Minimally Invasive Endoscopic Technology Equipment & Standards, Changsha 410008, China; 4 School of Materials Science and Engineering, Central South University, Changsha 410083, China

**Keywords:** pure iron, annealing, grain refinement, biodegradation, implant material

## Abstract

Biodegradable pure iron has gained significant interest as a biomedical material. For biodegradable implant applications, the biodegradation behavior of pure iron is important. In this work, the influence of ferrite grain size on the biodegradation rate for pure iron was studied by means of heat treatment that was annealed below the austenized temperature using as-forged pure iron. Grains were coarsened and a spectrum of ferrite grain sizes was gained by changing the annealed temperature. Biodegradation behavior was studied through weight loss tests, electrochemical measurements and microscopic analyses. Hardness (HV) and biodegradation rate (*P_i_* or *P_w_*) were linearly ferrite grain size-dependent: $\mathrm{HV}=58.9+383.2d^{-\frac{1}{2}}$, and $P_{i}=-0.023+0.425d^{-}{}^{\frac{1}{2}}$ or $P_{w}=0.056+0.631d^{-}{}^{\frac{1}{2}}$. The mechanism by which the role of grain size on biodegradation rate was attributed to the ferrite grain boundary traits.

## 1. Introduction

Biodegradable pure iron has recently gained significant interest in terms of its use in medical devices, such as bone scaffolds, fixtures and stents due to its good mechanical properties and biocompatibility in physiological environments, which provides sufficient temporary support to resist the applied load and effectively eliminates the potential risk of long-term complications through the progressive degradation in the body [1,2,3]. The degradable and absorbable functions for pure iron are highly desired because they do not need a secondary surgical procedure for the removal, which easily increases risk of the infection, surgical cost, and the likelihood of patient complications.

Work concerning the biodegradable implant applications of pure iron has involved in the conventional manufacturing procedures, such as microwave sintering, laser melting, casting and deformation processing [4,5,6,7]. Manufacturing processes play a fundamental role towards the biodegradation of pure iron. Zhao et al. comparatively studied the biodegradation behavior of pure iron prepared by microwave sintering (MS) and laser melting (LM) and found that the biodegradation rate of the MSed Fe was higher than that of the LMed Fe and their biodegradation rates were higher than that of the as-cast Fe [4]. Carluccio et al. [5] reported that the biodegradation rate of the pure iron manufactured via selective laser melting was over 50% higher than the corresponding cast equivalent. Bagherifard et al. [6] found that the inclined and multi-directional surface impacts accelerated the biodegradation of pure iron. Obayi et al. [7] studied the influence of the cross-rolling on the biodegradation of pure iron as biodegradable material for medical implants and found that the biodegradation was more uniform for the cross-rolled samples while the biodegradation rates between the cross-rolled samples and the straight-rolled samples did not show relevant differences in simulated body solution. Such conventional manufacturing methods often need further the post processing, such as annealing, to obtain the final product. It is known that the annealing of the metals has an important impact on the microstructural factors, such as grain szie, sub-structures, internal stresses, macro-segregations, impure inclusions and second phase particles, each of which might have an impact on the mechanical properties and the degradation response. The annealing of pure iron only needs reheating to a temperature below the austenized temperature for several hours, followed by air cooling. The mechanical properties and biodegradation behavior of pure iron are important for biodegradable implant applications. It is well established that the mechanical properties of pure iron were significantly influenced by annealing [8]. However, only little work has reported on the biodegradation behavior of pure iron upon annealing [9], and the details of grain growth upon annealing and its influence on biodegradation rate for pure iron is not yet fully understood, which restrains pure iron as biodegradable implant materials to meet the requirements of clinical applications. The biodegradation rate dependence of the grain size of pure iron is worthy of study. However, most work to date has only studied coarse and/or fine grain size but not a spectrum of grain size. 

In this work, pure iron was annealed at different annealing temperature to obtain a spectrum of ferrite grain size, and grain growth upon annealing and its influence on the biodegradation rate of pure iron was studied. The mechanism by which the role of the ferrite grain size on the biodegradation rate of pure iron was revealed. This work provides a reference to regulate the biodegradation behavior of pure iron through annealing and facilitates its use in biodegradable implant applications.

## 2. Experiments

**Figure 1:**
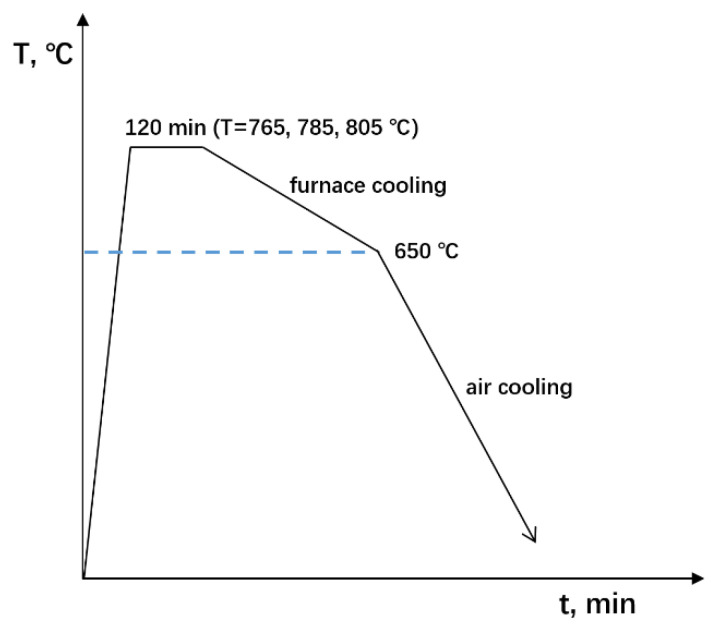
Schematic annealing procedure diagram.

Pure iron (provided by Institute of Metal Research, Chinese Academy of Science, Shenyang city, China) was used in this work at a high purity, with a chemical composition (wt.%) of 0.006 C, 0.002 Mn, 0.003 Si, 0.005 P, 0.005 S, 0.003 Ni, 0.002 Cr, 0.009 Ti, 0.002 Cu and Fe balance. Specimens of 10 mm diameter and 3 mm length were cut from the forged rods, which were hot-forged from the initial as-cast ingots. Annealing was performed for the forged specimens at 765, 785 and 805 °C for 2 h, followed by furnace cooling to 650 °C then air cooling, respectively. A schematic annealing procedure diagram is shown in Figure 1. Hereafter, three annealed conditions are designed as Fe765, Fe785 and Fe805, respectively. The microstructure was examined by optical microscopy by etching the mechanically polished surfaces of metallographic specimens using nitric acid alcohol with a 2% concentration. The microstructure was also characterized by electron back-scattered diffraction (EBSD). The hardness was tested using a digital Vickers indenter.

Potentiodynamic polarization test was conducted in Hank’s solution at 37 ± 0.5 °C through an Autolab system using a common three-electrode system with platinum as the counter electrode, saturated calomel as reference electrode (SCE) and the specimen as working electrode. The electrochemical impedance spectrum (EIS) was measured after open-circuit potential (OCP) had been stable for 3600 s. The biodegradation rate, *P_i_* (mm y^−1^), was calculated by Equation (1) using the corrosion current density measured, *i_corr_* (mA cm^−2^), from potentiodynamic polarization curves [10,11]:

(1) $$P_{i}=11.16i_{corr}$$

Immersion test was performed in Hank’s solution for 14 days at 37 ± 0.5 °C. The weight loss rate, Δ*W* (mg cm^−2^ d^−1^), was calculated by Equation (2), and the biodegradation rate, *P_w_* (mm y^−1^), was calculated by Equation (3), respectively [10,11].

(2) $$\Delta W=\left(W_{b}-W_{a}\right)/AT$$

(3) Pw=3.67ΔWD 

where *W_b_* and *W_a_* are the specimen mass before exposure and after exposure, respectively; *A* is the specimen area exposed to the corrosive solution (cm^2^); *T* is the time (d); and *D* is the density of the material in g/cm^3^ (7.87 g/cm^2^ for pure iron). Corroded surface morphology was observed using a scanning electron microscopy and corroded topographic map was characterized using a 3D-measuring laser microscope. 

## 3. Results

### 3.1. Grain Growth and Hardness Variation

**Figure 2:**
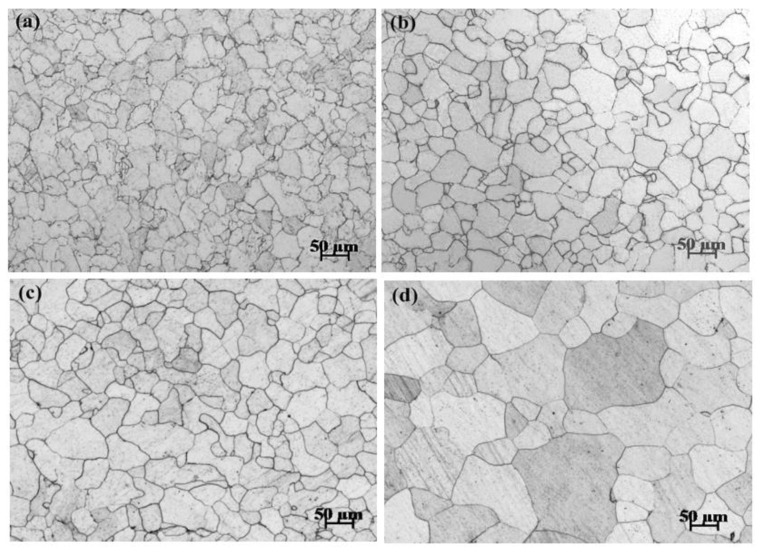
Optical micrographs at (**a**) the initial forged condition and (**b**–**d**) the different annealed conditions: (**b**) 765 °C, (**c**) 785 °C, (**d**) 805 °C.

Figure 2 shows the optical micrographs of the initial forged condition (Figure 2a) and the different annealed conditions (Figure 2b–d). There were no apparent inclusions or second phase particles in the matrix for all the microstructures. This indicated that the experimental pure iron was very clean and the influence of impure inclusions on its biodegradation was expected to be negligible. 

**Figure 3:**
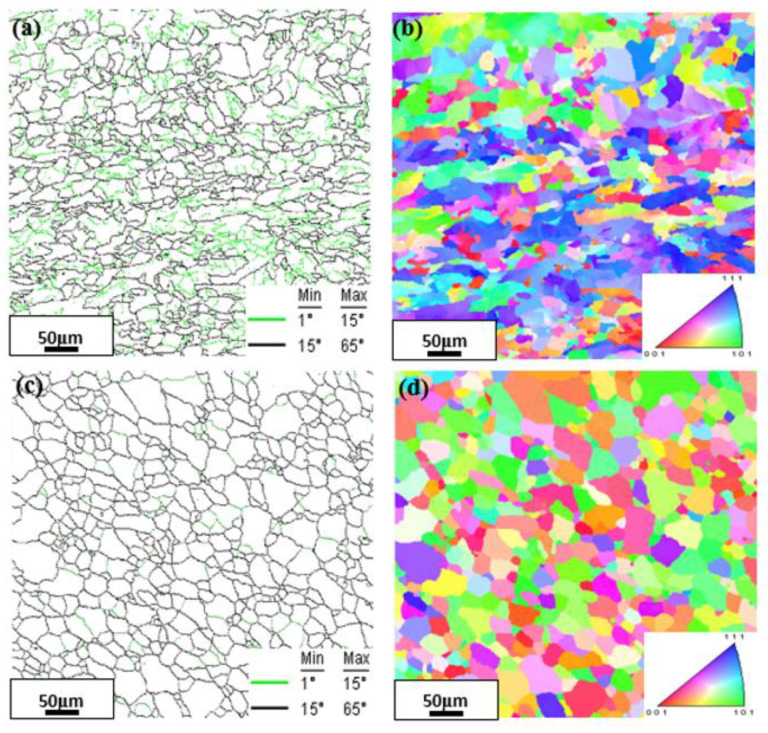
(**a**) the EBSD-characterized grain boundaries and (**b**) the IPF map of the initial forged condition; (**c**) the EBSD-characterized grain boundaries and (**d**) the IPF map of the representative annealed condition of Fe765.

As shown in Figure 2a, the initial forged condition presented some sub-structures or sub-grains in microstructure. Figure 3a,b shows the EBSD-characterized ferrite grain boundaries and the IPF map of the initial forged condition. The black lines indicated mis-orientations over 15° between the adjacent points (i.e., high angle grain boundary), and the green lines reflected the mis-orientations between 1 and 15° (i.e., low angle grain boundary). High angle grain boundaries were accompanied by large numbers of low angle grain boundaries. Therefore, the forged pure iron essentially accumulated a large number of sub-structures or sub-grains during deformation. When the forged pure iron was annealed, the nuclei were generated at the subgrain or grain boundaries of the original deformed microstructure, and the new undistorted ferrite grains with little residual stress and low dislocation density were formed, i.e., the growth of ferrite grains [12]. All the annealed microstructures consisted of quasi-polygonal ferrite grains, as shown in Figure 2b–d. Figure 3c,d shows the EBSD-characterized ferrite grain boundaries and the IPF map of the representative annealed condition of Fe765. The low angle grain boundaries almost disappeared in microstructure.

**Figure 4:**
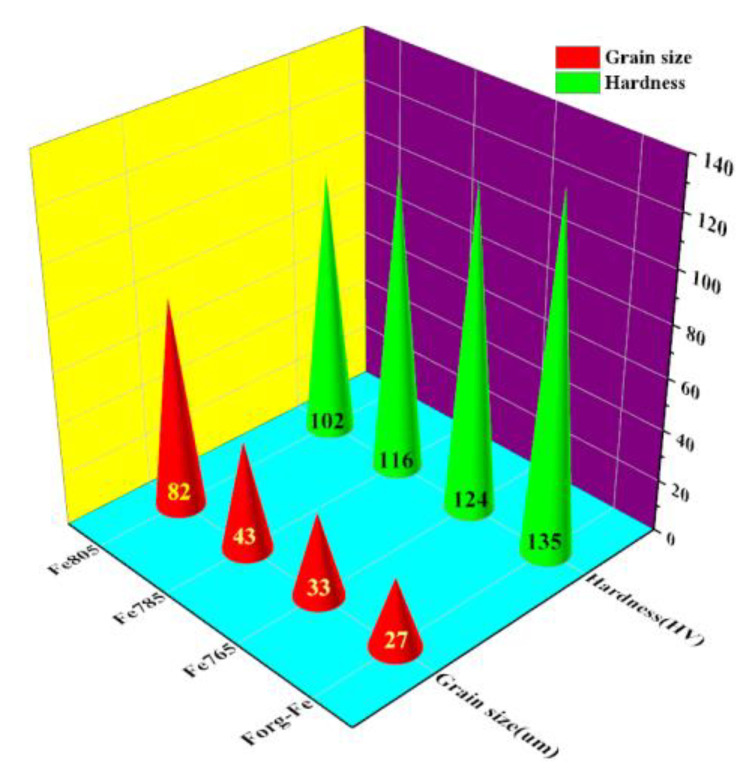
Average grain size and Vickers hardness at different annealed conditions (Fe765, Fe785, and Fe805), with the forged condition (Forg-Fe) as a comparison.

Figure 4 shows the average ferrite grain size in the initial forged condition and subsequent annealed conditions. Average ferrite grain size in the initial forged condition was 27 μm. A pronounced evolution of the ferrite grain size was presented when the annealed temperature changed. The driving force of the regenerated undistorted ferrite grains became higher at a higher annealed temperature, forming the new undistorted larger grains. As annealed at 765 °C, average ferrite grain size was 33 μm. The increasing annealed temperature caused the coarsening of the ferrite grain size. As annealed at 785 °C and 805 °C, the average ferrite grain size was 43 μm and 82 μm, respectively. Figure 4 also depicts the Vickers hardness values of the forged and different annealed conditions. For the forged condition, the hardness was 134.6 HV. The hardness changed when the annealed temperature changed. As annealed at 765 °C, the hardness was 123.9 HV. As annealed at 785 °C and 805 °C, the hardness was 116.1 HV and 102.2 HV, respectively. The hardness decreased with the increase of the annealed temperature. The ferrite grain size increased with the increase of the annealed temperature, as mentioned above. Therefore, the hardness decreased with increasing the ferrite grain size. The grain boundaries blocked the dislocation movement. A smaller grain size presented a larger grain boundary proportion, with a more obvious hindrance [13]. The regression equation that described the relationship between ferrite grain size and hardness was obtained as Equation (4) had a correlation coefficient in 0.99, using all the measured data.

(4) $$\mathrm{HV}=58.9+383.2d^{-\frac{1}{2}}$$

The hardness value of pure iron was linearly ferrite grain size-dependent, following a so-called Hall–Petch relation [14].

### 3.2. Biodegradation

**Figure 5:**
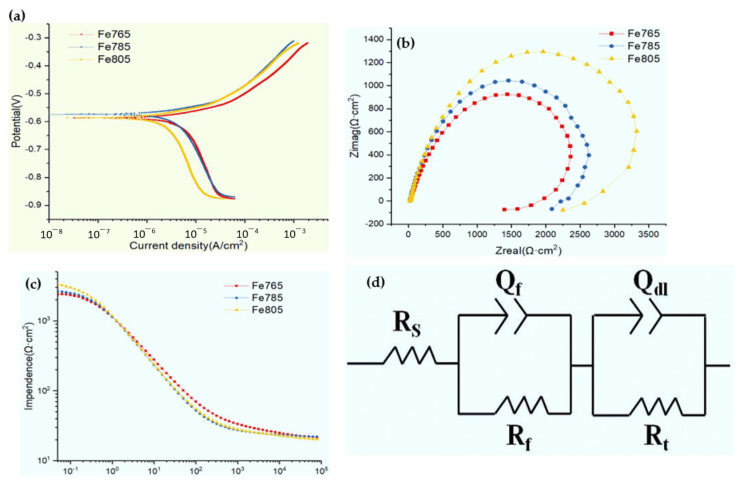
(**a**) Polarization curves, (**b**) Nyquist plots and (**c**) Bode plots of the different annealed conditions in Hank’s solution and (**d**) the equivalent circuit for impedance data fitting.

Figure 5a depicts potentiodynamic polarization curves for the specimens at different annealed temperatures in Hank’s solution. The corrosion potential (*E_corr_*) value had little change for the specimens at different annealed temperature. The *E_corr_* value can reflect the thermodynamic information of the corrosion and can thus reflect the biodegradable tendency. The *E_corr_* value of the metal is related to such internal states as the residual stress, the deformed twins and texture, etc [15]. A similar *E_corr_* value for the specimens at different annealed temperatures in the present work indicated that there had been a similar internal state, which means that the designed annealed conditions excluded the effects of other structure factors from the processing, such as sub-structures, internal stresses, residual stress, twins and texture. The *i_corr_* values of the specimens at the different annealed temperature had relatively obvious differences, which were measured from the linear cathodic branch of polarization potential curves according to Tafel extrapolation and are shown in Table 1. The *i_corr_* value decreased with the increase of the annealed temperature.

Figure 5b,c shows the electrochemical impedance spectra (EIS) data of the specimens at the different annealed temperature in Hank’s solution. The shape of the Nyquist plots are similar for the specimens at the different annealed temperature, with one single capacitive loop in the measured frequency range. The corresponding capacitive loop diameter was involved in the anti-polarization. A larger capacitive loop diameter reflected a lower corrosion rate [16,17]. The diameter size order increased with the increase of the annealed temperature. The equivalent circuit that can be used to study the Nyquist spectra is illustrated in Figure 5d. The corresponding results are shown in Table 2, in which R_t_ is the charge transfer resistance, R_s_ is the solution resistance, CPE_dl_ is the double-layer capacitor, R_f_ is the film resistance and CPE_f_ is the capacitance that reflects the effect of the surface film. The R_t_ value order is Fe805 > Fe785 > Fe765, corresponding to the size order of the above-mentioned EIS spectra diameter.

**Figure 6:**
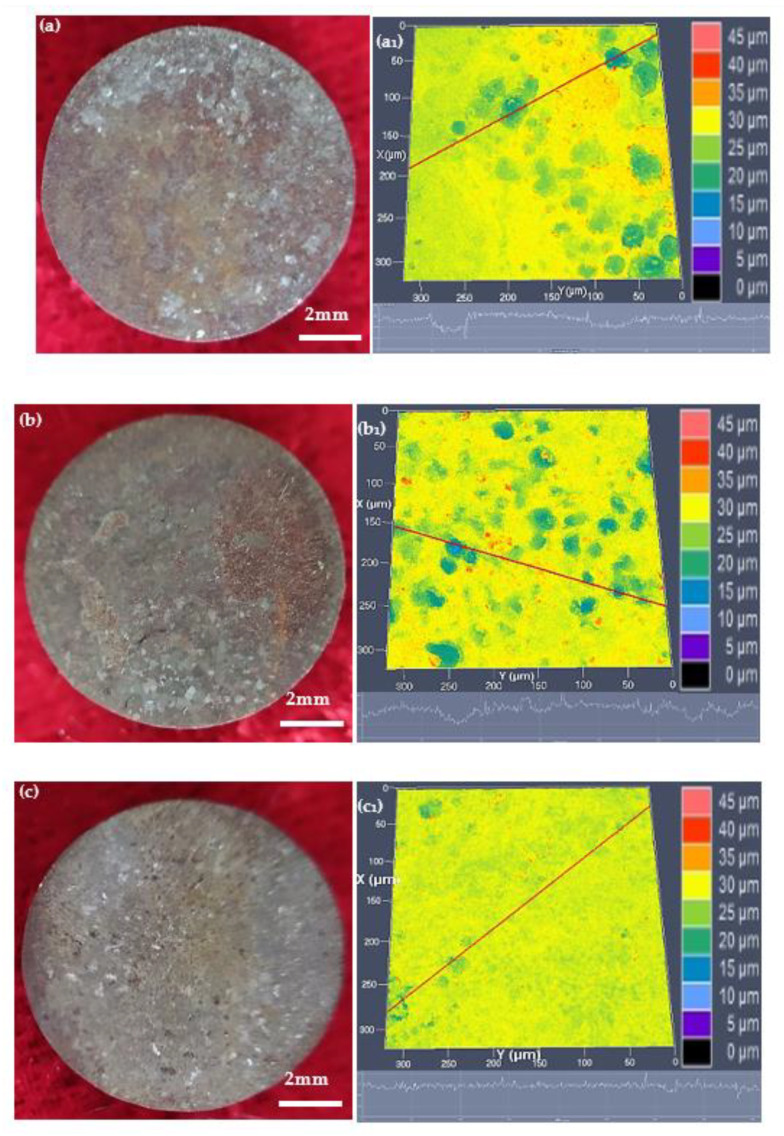
Surface morphologies ((**a**–**c**)) and topographic maps ((**a**_1_–**c**_1_)) of the different annealed conditions: (**a**,**a**_1_) for annealed at 765 °C, (**b**,**b**_1_) for annealed at 785 °C, (**c**,**c**_1_) for annealed at 805 °C.

Figure 6 shows the corroded surface morphologies and topographic maps of the specimens at the different annealed temperatures after removing the products of the biodegradation, which were immersed in Hank’s solution for 14 days. The corroded surfaces were relatively intact and compact, and the dimly discernible biodegradation became lighter with the increase of the annealed temperature, as shown in Figure 6a–c. The corroded topographic maps, as shown in Figure 6a_1_–c_1_, agreed with the corresponding corroded surface morphologies. There were some preferential small and shallow pits when annealed at 765 °C and 785 °C (Figure 6a_1_,b_1_), while there was relatively more uniform on a macro-scale when annealed at 805 °C (Figure 6c_1_).

Table 3 shows the biodegradation rate, *P*_i_, of the specimens at different annealed temperatures that were calculated by Equation (1) using the measured *i_corr_* data, and the biodegradation rate, *P_w_*, of the specimens at the different annealed temperatures that were calculated by Equation (2) and Equation (3) using the weight loss data. The corresponding initially forged data were also listed in Table 3 as the controls. The *P*_i_ value or the *P*_w_ value of the initial forged condition was relatively high. Those of the specimens at the different annealed temperature decreased with the increase of the annealed temperature.

## 4. Discussion

### 4.1. Grain Growth upon Annealing

Ferrite grain growth upon annealing is a cannibalistic process. The growth of some grains must be accommodated by the shrinkage and ultimate disappearance of other grains. The increase of the average ferrite grain size decreases the area of grain boundary and thus decreases the energy of grain boundary. For pure iron, the circumstance of the grain grown upon annealing is almost entirely decided by the decrease of grain boundary energy, because the influence of other structural factors, such as impure inclusions, macro-segregations and second phase particles, may be negligible. The EBSD characterized ferrite grain boundaries well described the new undistorted ferrite grains with little residual stress and low dislocation density upon annealing, i.e., the growth of ferrite grains. Therefore, the driving force of the ferrite grain growth is the reduction of the grain boundary energy [18], indicated as:

(5) $$F_{d\;}=2\frac{\gamma }{R}$$

where *F_d_* is the driving force of grain growth, *γ* is grain boundary energy and *R* is grain size.

From a thermo-dynamic viewpoint, the grain growth kinetic is assumed to obey an Arrhenius relation:

(6) dRdt=AFdexp(−Q/KT)

where *A* is constant, *Q* is the activation energy of grain growth, *k* is gas constant and *T* is the absolute temperature.

Substituting Equation (5) into Equation (6)

(7) dRdt=2Aγexp(−Q/kT)/R

Based on some calculus relation assumptions

(8) $$R^{2}=4A\gamma \left(t-C\right)exp\left(-Q/kT\right)$$

where *C* is calculus constant. Therefore, the ferrite grain size increases with the increasing annealed temperature, which is substantiated by the present results, as depicted in Figure 2.

### 4.2. Biodegradation

**Figure 7:**
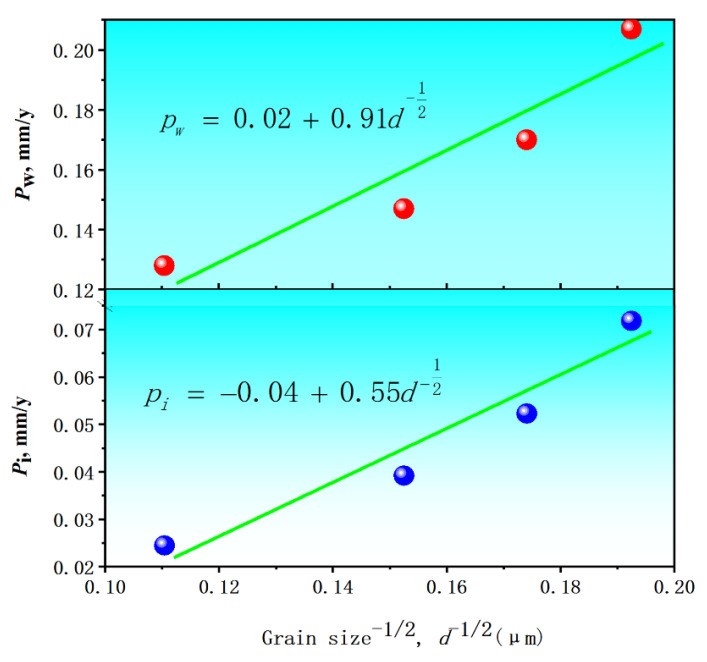
The relation between the biodegradation rate (*P_i_* or *P_w_*) and the average ferrite size (*d*).

The biodegradation rate of the specimens at the different annealed temperatures in Hank’s solution was different, as stated above. This difference in biodegradation rate was primarily due to the evolution of ferrite grain size caused by annealing. The evolution of the ferrite grain size produced the change of the ferrite grain boundary area. The micro-segregation of the ferrite grain boundary occurred in pure iron (99.9%). The standard electrode potential of iron is −0.44 V, which is usually higher than the main metal impurities in pure iron, such as Cr (−0.74), Mn (−1.179), Ti (−1.630) and Al (−1.663). Therefore, the potential at grain boundary with more metal impurities is lower than that of the pure iron matrix, and iron acts as cathode and grain boundary as anode. Reducing grain size is equivalent to increasing grain boundary area [19]. At this time, the biodegradation resistance decreases with decreasing grain size, because the biodegradation resistance of the grain boundary is low and the increase of grain boundary area reduces the biodegradation resistance. Therefore, the ferrite grain boundary largely influences the biodegradation of pure iron. Furthermore, the ferrite grain size (*d*) increased with increasing the annealed temperature, while the biodegradation rate (*P_i_* or *P_w_*) decreased with increasing the annealed temperature. Figure 7 depicts the plots of *P_i_* versus *d*^−1/2^ and *P_w_* versus *d*^−1/2^, in which the initial forged condition is also used as a control. The obtained plots synchronized well to achieve a monotonic character. The relationship between the *P*_i_ and the *d* was expressed as Equation (9) with a correlation coefficient of 0.97 and that between the *P_w_* and the *d* was expressed as Equation (10) with a correlation coefficient of 0.94. As a result, the biodegradation rate of pure iron was linearly ferrite grain size-dependent, following a so-called Hall–Petch relation [14].

(9) $$P_{i}=-0.04+0.55d^{-}{}^{\frac{1}{2}}$$

(10) $$P_{w}=0.02+0.91d^{-}{}^{\frac{1}{2}}$$

### 4.3. Safe Intake and Toxicity

Acceptable safe intake and toxicity is essential for a biodegradable implant metal, which mainly relies on the biodegradation product toxicity [20], the released concentration of the metal ions [21], and the metal ions themselves against the cell metabolic activities [22]. As the biodegradation of pure iron occurs, the anodic reaction is expressed as: $\mathrm{Fe}-2e\to {\mathrm{Fe}}^{2+}$ and the cathodic reaction is expressed as: $2H_{2}O+O_{2}+4e\to 4{\mathrm{OH}}^{-1}$. Therefore, $\mathrm{Fe}{\left(\mathrm{OH}\right)}_{2}$ is obtained according to ${\mathrm{Fe}}^{2+}+2{\mathrm{OH}}^{-1}\to 2\mathrm{Fe}{\left(\mathrm{OH}\right)}_{2}$ and subsequently the obtained $\mathrm{Fe}{\left(\mathrm{OH}\right)}_{2}$ is easily oxidized to form $\mathrm{Fe}{\left(\mathrm{OH}\right)}_{3}$ due to its instability according to $4\mathrm{Fe}{\left(\mathrm{OH}\right)}_{2}+2H_{2}O+O_{2}\to 4\mathrm{Fe}{\left(\mathrm{OH}\right)}_{3}$. Furthermore, the Ca/P compounds usually precipitate on the hydroxide layer surface as the biodegradation proceeds [23]. All these biodegradation products are nontoxic [24]. The concentration of the released iron ions immersed in Hank’s solution for 24 h was 2.52 μg/(mL day) for the Fe765, which was the maximum in the present annealed conditions due to its relatively high biodegradation rate. Iron as an element is essential for the human body. $\mathrm{Fe}{\left(\mathrm{OH}\right)}_{3}$ or Fe^3+^ was produced during the biodegradation as described above. Fe^3+^ binds to the transferrin exclusively, and subsequently the iron-loaded transferrins are transported to the cell surface, where the iron ion is absorbed due to the endocytosis [25]. The average daily released iron ion concentration of Fe765 is maximum in the present annealed conditions and is 2.52 μg/(mL day) as mentioned above, much lower than the value of the iron half-maximal inhibitory concentration (IC50) [21]. As reported, an iron ion concentration of less than 10 μg/(mL day) had a favorable influence on the endothelial cell metabolic activity and less than 50 μg/(mL day) had little inhibitory influence on the endothelial cell metabolic activities [26]. Therefore, the biocompatibility of the pure iron experiencing annealing is acceptable.

## 5. Conclusions

1. As annealed, the nuclei were generated at the sub-grain or grain boundaries of the original deformed microstructure, and the new undistorted ferrite grains with little residual stress and low dislocation density were formed.
2. A spectrum of ferrite grain size was gained by changing the annealed temperature. Hardness (HV) and biodegradation rate (*P_i_* or *P_w_*) were linearly ferrite grain size-dependent:
3. $\mathrm{HV}=58.9+383.2d^{-\frac{1}{2}}$; and $P_{i}=-0.04+0.55d^{-}{}^{\frac{1}{2}}$ or $P_{w}=0.02+0.91d^{-}{}^{\frac{1}{2}}$.
4. The biocompatibility of the pure iron experiencing annealing is acceptable.
5. The obtained results were quite helpful for better regulating the biodegradation of biodegradable pure iron through annealing.

## Figures and Tables

**Table 1:** Polarization data of the different annealed conditions in Hank’s solution.

Specimens No.	*E_corr_* (V_SCE_)	*i_corr_* (μA/cm^2^)
Fe765	−0.587	4.686
Fe785	−0.574	3.514
Fe805	−0.586	2.196

**Table 2:** Parameters obtained from the simulation circuit in Hank’s solution.

Specimen No.	R_S_ (Ω cm^2^)	CPEfilm-T (Ω^−1^ s^−n^/cm^2^)	n	R_f_ (Ω cm^2^)	CPEct-T (Ω^−1^ s^−n^/cm^2^)	n	R_t_ (Ω cm^2^)
Fe765	20.47	6.339 × 10^−5^	0.692	15.11	1.275 × 10^−4^	0.724	2.822 × 10^3^
Fe785	21.79	1.59 × 10^−4^	0.708	14.38	6.18 × 10^−6^	0.883	3.40 × 10^3^
Fe805	19.62	4.272 × 10^−5^	0.747	8.964	1.335 × 10^−4^	0.775	4.915 × 10^3^

**Table 3:** Biodegradation rate of the initial forged and different annealed conditions in Hank’s solution.

Specimen No.	*P_i_* (mm y^−1^)	*P_w_* (mm y^−1^)
Initial forged condition	0.0718	0.207 ± 0.6
Fe765	0.0523	0.170 ± 0.5
Fe785	0.0392	0.147 ± 0.3
Fe805	0.0245	0.128 ± 0.3

## Data Availability

Not applicable.
